# Diamond–Blackfan anemia *RPL35A*: a case report

**DOI:** 10.1186/s13256-019-2127-3

**Published:** 2019-06-18

**Authors:** Colin Byron Noel

**Affiliations:** Department of General / Transplant Surgery, University of Cape Town and Groote Schuur Hospital, J-Floor, Old Main Building, Observatory, Cape Town, 7925 South Africa

**Keywords:** Diamond–Blackfan anemia, DBA, Neutropenia, Bone marrow failure, Rare disease, *RPL35A*

## Abstract

**Background:**

Diamond–Blackfan anemia is a rare congenital red blood cell aplasia characterized by failed erythropoiesis, congenital abnormalities in up to 50% of patients, growth retardation in up to 30% of patients, and a predisposition to malignancy. Diamond–Blackfan anemia is both clinically and genetically a heterogenous condition ranging from subtle asymptomatic erythroid abnormalities to non-immune hydrops fetalis. Current treatment options include corticosteroid therapy, chronic red blood cell transfusions, and hematopoietic stem cell transplantation with gene therapy receiving recent attention. We report the first documented case of Diamond–Blackfan anemia in a Caucasian girl secondary to a sporadic heterozygous whole gene deletion in *RPL35A* in South Africa. Limited resources, non-availability of tests, unfamiliarity that comes with rare diseases, an expanded differential diagnosis, and an associated neutropenia led to a delay in the diagnosis of Diamond–Blackfan anemia. This case reminds clinicians of Diamond–Blackfan anemia as a cause of aplastic anemia and highlights the difficulty and obstacles in diagnosing Diamond–Blackfan anemia in resource-limited countries.

**Case presentation:**

We report a case of a 6-week-old Caucasian girl presenting with urosepsis and heart failure secondary to a severe anemia and neutropenia. Limited experience and resources resulted in a delay in diagnosis. Genetic studies later confirmed a heterozygous whole gene deletion of *RPL35A.* Initial treatment was directed toward correcting the anemia with red blood cell transfusion every 3 to 5 weeks.

**Conclusion:**

Diamond–Blackfan anemia is a rare disease that carries significant morbidity and mortality if not diagnosed early and managed appropriately. Limited health resources, patient registries, and specialists as seen in developing countries result in a paucity of knowledge about Diamond–Blackfan anemia in Africa. This case reminds clinicians about Diamond–Blackfan anemia as a cause for anemia in infants, the limitations in making the diagnosis in under-resourced health care systems, and the need for standardized treatment protocols applicable to resource-limited countries.

## Background

Diamond–Blackfan anemia (DBA) is a rare congenital red blood cell (RBC) aplasia characterized by failed erythropoiesis, congenital abnormalities, and a predisposition to malignancy. Also known as one of the rare groups of inherited bone marrow failure syndromes, this clinically and genetically heterogeneous condition was first described by Josephs in 1936 and then as a distinct entity in 1938 by Diamond and Blackfan [1].

Numerous theories regarding the etiology of DBA have been proposed. Traditionally, DBA was considered a ribosomopathy caused by genetic mutations in one of 19 ribosomal proteins affecting ribosome synthesis. More recently, however, three non-ribosomal gene mutations have been identified: *GATA1* (an erythroid transcription factor), *TSR2* (a gene encoding a direct binding partner of *RPS26*) [2, 3], and *EPO* [4]. The exact pathogenesis underlying the tissue expression in DBA and predilection for erythroid defects is not fully understood.

The characteristic hematological features of DBA include a severe normochromic macrocytic anemia, reticulocytopenia, isolated erythroid hypoplasia in the bone marrow, and an increased erythrocyte adenosine deaminase (eADA) [5], erythrocyte “i” antigen, and fetal hemoglobin (Hb) [6].

The prevalence of DBA in South Africa is not currently known. Data published from international registries report an incidence of 1:100,000–200,000 live births [7, 8]. With no known geographical variance in DBA and approximately 1,000,000 live births annually in South Africa, one can estimate between five and ten cases of children born with DBA every year. Limitations in access to children’s health care and knowledge around rare diseases remain a significant challenge in developing countries and could explain the low incidence of reported cases. Although the anemia can present at any age, the median age of diagnosis is 12 weeks of age, with 90% of patients presenting with anemia within the first year [9]. It is therefore essential that DBA be considered in the workup of any infant presenting with anemia. We present the first known recorded case of DBA in South Africa.

## Case presentation

A 6-week-old Caucasian girl was admitted to a private hospital in South Africa with acute onset symptomatic cardiac failure secondary to anemia. Her parents reported a 1-day history of lethargy, poor feeding, shortness of breath, and irritability on a background history of progressive pallor.

There was no family history of note. Antenatal history included a low maternal pregnancy-associated plasma protein A (PAPP-A) level (0.376 IU/L) which resulted in a high-risk screening protocol for intrauterine growth restriction (IUGR) and fetal chromosomal anomalies. Cell-free fetal deoxyribonucleic acid testing from maternal blood excluded aneuploidies for the common trisomies [10–12] and subsequent fetal anomaly ultrasound and echocardiogram scans were all normal. A caesarean section was performed at 37 weeks for spontaneous labor, IUGR, and breech presentation. The delivery was uneventful and apart from a low birth weight of 2465 g, a healthy baby was discharged 3 days post caesarean section as per normal protocol.

On admission to hospital at 6 weeks of age, the baby under examination was severely anemic, tachycardic, and lethargic. There were no stigmata of immunocompromise, infection, or icterus. The baby weighed 3200 g with a head circumference of 38 cm.

There were no obvious craniofacial or skeletal abnormalities of note and examinations of her other systems were normal. The preliminary results with normal range for age in brackets showed a hemoglobin (Hb) level of 3.1 gm/dL (10–18 gm/dL) and a hematocrit of 9% (31–55%), mean corpuscular volume of 106 fl (85–123 fl), mean corpuscular Hb concentration 34 g/dL (32–37 g/dL), reticulocyte production index of 0.0, and an absolute reticulocyte count of 5.1 × 10^9^/L (20–60 × 10^9^/L). Her white cell count was low 4.0 × 10^9^/L (5–19.5 × 10^9^/L) but apart from a low neutrophil count of 0.32 × 10^9^/L (1–9 × 10^9^/L), the remaining differential count was normal. Her platelet count was increased 655 × 10^9^/L (140–420 × 10^9^/L). Her C-reactive protein was marginally raised at 7.7 mg/L (< 5 mg/L), and the infective work up was positive for *Escherichia coli* cultured from the urine. Tests for cytomegalovirus, human immunodeficiency virus (HIV), rubella, Epstein–Barr virus, toxoplasmosis, herpes simplex virus 1 and 2, and parvovirus B19 were all negative. A diagnosis of *E. coli* urosepsis was made. The baby was transfused with leukodepleted irradiated red cell concentrate to an Hb level of 10 g/dL and given goal-directed antibiotics and discharged 6 days later.

**Fig. 1:**
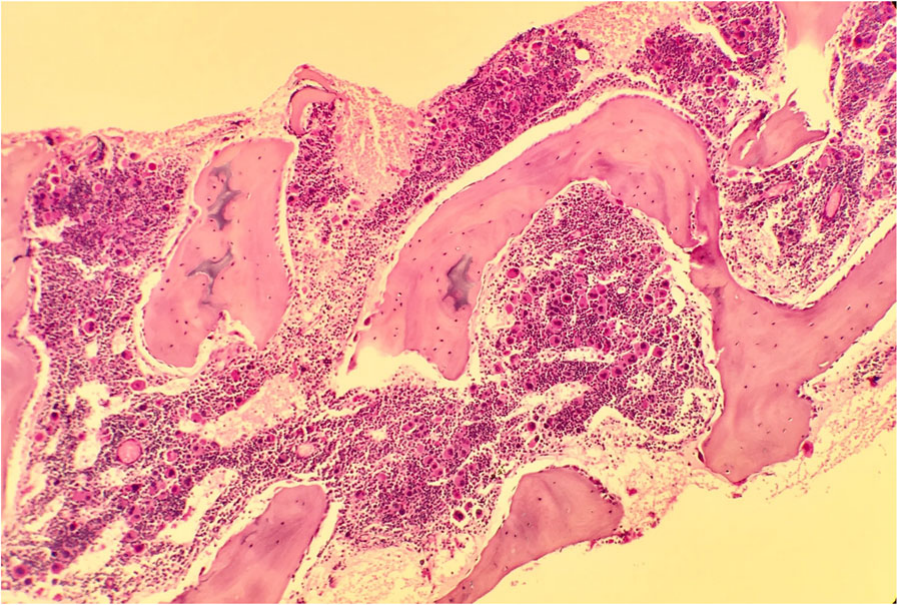
Bone marrow trephine hematoxylin and eosin stain. Normocellular trephine showing normal megakaryopoiesis and granulopoiesis

**Fig. 2:**
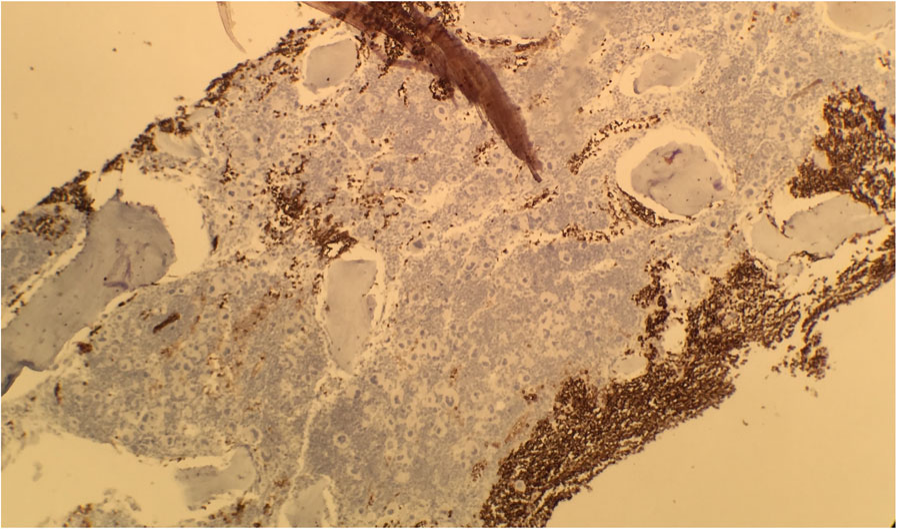
Bone marrow glycophorin. Absence of red blood cell precursors

Readmission 14 days later with an anemia (Hb 7.7 g/dL) and associated reticulocytopenia of 7.0 × 10^9^/L (20–60 × 10^9^/L) prompted a provisional diagnosis of transient erythroblastopenia of childhood (TEC), which was made after infection, HIV, and tuberculosis were excluded. Three further admissions over the next 3 months for anemia requiring red cell transfusions and a persistent neutropenia prompted a bone marrow biopsy (Figs. 1 and 2).

Review of the bone marrow biopsy showed reactive features with markedly increased megakaryopoiesis and significant lymphocytic infiltrate. Flow cytometry demonstrated the infiltrate to consist of T cells, mature B cells, and hematogones. An absence of red cell precursors and immunohistochemical glycophorin stain on the bone marrow trephine confirmed a pure red cell aplasia.

Due to the unavailability of molecular and eADA testing in South Africa, specimens for molecular testing were sent to Oxford, UK, for identification of a possible heterozygous pathogenic variant in one of the genes associated with DBA. A multigene panel using the Multiplex Ligation-dependent Probe Amplification kit (MRC-Holland) confirmed a heterozygous whole gene deletion of *RPL35A*. In addition, dried plasma was sent to Duke University Medical Center, USA, to exclude adenosine deaminase 2 deficiency.

Once the diagnosis of DBA was made, the non-standardized management and limited experience in managing DBA in South Africa resulted in inconsistencies in opinion in the optimal early management of the case. Controversies around Hb transfusion threshold, optimal Hb target, frequency of transfusions, and timing and dosage of corticosteroid treatment and hematopoietic stem cell transplantation (HSCT) resulted in different opinions from different specialist practitioners.

Together with input from international DBA specialists managing large numbers of patients with DBA, the initial hematopoietic management in this case was directed toward correcting the anemia with transfusions every 3–5 weeks with irradiated leukodepleted RBC concentrate. A transfusion threshold of 8 g/dL was used and a volume of 10–15 ml/kg transfused on each visit. Planned iron chelation therapy to prevent transfusional hemosiderosis will be delayed until approximately 170–200 ml/kg of transfused red packed cells has been given. A planned trial of corticosteroids will be given at 1 year of age.

At follow-up at 6 months of age, the baby was stable requiring red cell concentrate infusion therapy every 3–4 weeks. Persistence in the neutropenia was noted, with no changes in the other cell lineages. Her current ferritin level is 573 μg/L and a total of 90 ml/kg of red packed cells has been transfused, thus, iron chelation therapy has not yet been instituted. Apart from a delay in gross motor development and growth (weight and height), all other parameters and development are within normal limits.

## Discussion

DBA is a rare cause of aplastic anemia and the main differential diagnosis of anemia due to decreased RBC production includes TEC, infections, and other genetic causes of bone marrow failure. These are, however, usually associated with additional cytopenias and include Shwachman–Diamond syndrome, Fanconi’s anemia, dyskeratosis, and Pearson marrow pancreas syndrome. Other acquired causes include aplasia associated with viral infections, drugs, autoimmune conditions, malignancies, and, rarely in adults, a thymoma should be considered [13].

*RPL35A* is a gene that encodes a 60S large ribosomal subunit protein and accounts for 3.3% of cases of DBA identified with a gene mutation [6]. It was described by Farrar *et al.* [14] in 2008 and was the first large ribosomal subunit protein defect directly linked to DBA in humans. Although a pure red cell aplasia is typical of DBA, *RPL35A* mutations may have an associated neutropenia [9]. To date, mutations in 19 ribosomal genes (*RPS7, RPS10, RPS15A, RPS17, RPS19, RPS24, RPS26, RPS27, RPS28, RPS29, RPL5, RPL11, RPL15, RPL18, RPL26, RPL27, RPL31, RPL35, RPL35A*) and 3 non-ribosomal genes (*GATA1, TSR2, EPO*) have been implicated in DBA [9].

The criteria used to establish the diagnosis of DBA have evolved. The current criteria proposed by Vlachos *et al.* [8] use clinical, histological, biochemical, and genetic parameters. Using the current proposed guidelines, there are a number of obstacles to making the diagnosis of DBA in South Africa and other countries in Africa. This is largely due to limited resources, tests, and health care services available. In addition, the historical access to health care and worker migration may influence obtaining an accurate family history about inheritance patterns of diseases. In children, TEC is the main differential diagnosis which must be excluded before making the diagnosis of DBA. Both i-RBC antigen testing and eADA activity testing which are used to differentiate TEC from DBA are not freely available in Africa. In addition, both genetic testing for DBA and eADA are not available in South Africa, and although bone marrow biopsies and histopathology are available, it is limited to a few of the larger centralized health institutions.

DBA currently has no curative treatment option. Mainstay treatment options to correct the anemia include chronic RBC transfusions, corticosteroid therapy, and HSCT [10, 15, 16]. Gene therapy, although still novel, does provide a theoretical cure to DBA. Increasing interest in gene therapy using viral vectors [6, 17] and novel gene editing tools CRISPR/Cas9 [18] could change current treatment protocols. Debnath *et al.* [6] recently reported successfully correcting the anemia in a mouse with DBA using a lentiviral vector with cellular promoters.

Current guidelines, however, are to initially treat the anemia with red cell transfusions until 1 year of age when a trial of steroids should be done [15]. Some authorities propose an earlier trial of steroids (6 months of age) in certain cases such as the possibility of no access to safe blood products. There are, however, concerns around growth stunting and neuromotor development with early use of corticosteroids [19, 20]. Despite 70–80% of patients initially responding to corticosteroids, only half remain on corticosteroid doses low enough to minimize toxicity [10]. Approximately 20% go into remission and the remaining 40% remain transfusion dependent [11]. Apart from corticosteroids, there are limited case reports of remission or improvement in Hb level using leucine [13, 21] or metoclopramide [12].

## Conclusion

DBA although a rare cause for red cell aplasia needs to be considered in the differential diagnosis especially in infants. The diagnosis of DBA in this patient subsequently highlighted not only the difficulty in diagnosing DBA in a resource-limited setting, but also the lack of consensus in treating DBA. This case highlights the need for the establishment of a protocolized workup for infants with persistent anemia of unknown cause that includes rare genetic causes. Limited access to investigations needed to make the diagnosis of DBA using current diagnostic criteria needs to be reviewed due to the inaccessibility in Africa of some of the specialized tests. Familiarization with international treatment guidelines is important in the treatment of rare diseases but local guidelines need to be established.

Lastly, this case highlights the paucity of knowledge around DBA in Africa and the need for further research into the epidemiology of DBA in Africa and the establishment of local treatment guidelines.

## Data Availability

The datasets used and/or analyzed during the current study are available from the corresponding author on reasonable request.

## Abbreviations

DBA: Diamond–Blackfan anemia
eADA: Erythrocyte adenosine deaminase
Hb: Hemoglobin
HIV: Human immunodeficiency virus
HSCT: Hematopoietic stem cell transplantation
IUGR: Intrauterine growth restriction
RBC: Red blood cell
TEC: Transient erythroblastopenia of childhood
